# A numerical similarity approach for using retired Current Procedural Terminology (CPT) codes for electronic phenotyping in the Scalable Collaborative Infrastructure for a Learning Health System (SCILHS)

**DOI:** 10.1186/s12911-015-0223-x

**Published:** 2015-12-11

**Authors:** Jeffrey G. Klann, Lori C. Phillips, Alexander Turchin, Sarah Weiler, Kenneth D. Mandl, Shawn N. Murphy

**Affiliations:** Harvard Medical School, Boston, MA USA; Partners Healthcare, Boston, MA USA; Massachusetts General Hospital, Boston, MA USA; Brigham and Women’s Hospital, Boston, MA USA; Harvard Clinical Research Institute, Boston, MA USA; Computational Health Informatics Program, Boston Children’s Hospital, Boston, MA USA

**Keywords:** CPT codes, Biomedical ontologies, Medical informatics, Phenotype, Data mining/methods, Biomedical research/methods, Electronic health records/trends

## Abstract

**Background:**

Interoperable phenotyping algorithms, needed to identify patient cohorts meeting eligibility criteria for observational studies or clinical trials, require medical data in a consistent structured, coded format. Data heterogeneity limits such algorithms’ applicability. Existing approaches are often: not widely interoperable; or, have low sensitivity due to reliance on the lowest common denominator (ICD-9 diagnoses). In the Scalable Collaborative Infrastructure for a Learning Healthcare System (SCILHS) we endeavor to use the widely-available Current Procedural Terminology (CPT) procedure codes with ICD-9. Unfortunately, CPT changes drastically year-to-year – codes are retired/replaced. Longitudinal analysis requires grouping retired and current codes. BioPortal provides a navigable CPT hierarchy, which we imported into the Informatics for Integrating Biology and the Bedside (i2b2) data warehouse and analytics platform. However, this hierarchy does not include retired codes.

**Methods:**

We compared BioPortal’s 2014AA CPT hierarchy with Partners Healthcare’s SCILHS datamart, comprising three-million patients’ data over 15 years. 573 CPT codes were not present in 2014AA (6.5 million occurrences). No existing terminology provided hierarchical linkages for these missing codes, so we developed a method that automatically places missing codes in the most specific “grouper” category, using the numerical similarity of CPT codes. Two informaticians reviewed the results. We incorporated the final table into our i2b2 SCILHS/PCORnet ontology, deployed it at seven sites, and performed a gap analysis and an evaluation against several phenotyping algorithms.

**Results:**

The reviewers found the method placed the code correctly with 97 % precision when considering only miscategorizations (“correctness precision”) and 52 % precision using a gold-standard of optimal placement (“optimality precision”). High correctness precision meant that codes were placed in a reasonable hierarchal position that a reviewer can quickly validate. Lower optimality precision meant that codes were not often placed in the optimal hierarchical subfolder. The seven sites encountered few occurrences of codes outside our ontology, 93 % of which comprised just four codes. Our hierarchical approach correctly grouped retired and non-retired codes in most cases and extended the temporal reach of several important phenotyping algorithms.

**Conclusions:**

We developed a simple, easily-validated, automated method to place retired CPT codes into the BioPortal CPT hierarchy. This complements existing hierarchical terminologies, which do not include retired codes. The approach’s utility is confirmed by the high correctness precision and successful grouping of retired with non-retired codes.

## Background

Medical data in structured, coded format allows interoperable data analysis with consistent meaning. This is essential for next-generation electronic phenotyping algorithms, which find patients with specific diseases using electronic health record (EHR) data. For example, clinical data research networks which are part of the National Patient-Centered Clinical Research Network (PCORnet) are seeking to identify cohorts of patients with both rare and common diseases in a harmonized federated dataset across scores of institutions. PCORnet is an initiative funded by PCORI to create a nationwide research infrastructure [1].

Unfortunately, EHR data are complex and data consistency and integrity is highly variable between clinics and across EHR platforms [2]. Existing clinical quality measures published by the National Quality Forum (NQF), for example, correlate poorly with data actually in EHR systems [3, 4]. Perhaps for this reason, the Agency for Healthcare Research and Quality (AHRQ)’s Patient Safety Indicator (PSI) initiative has relied entirely on ICD-9 billing codes. But the AHRQ PSIs have been shown to have low sensitivity in detecting adverse events (as low as 10 % in some cases) [5]. This is likely due to the low correlation of billing diagnoses with actual disease. A review of phenotyping studies found that combining ICD-9 codes with other data sources improves predictive ability [6]. However, heterogeneity of data increases as one moves beyond data generated for medical claims. Therefore, approaches relying on billing-generated data are likely to be more portable than others.

Procedure codes are an important component of claims-related data. In the US, procedures are most commonly coded in the Current Procedural Terminology (CPT) format. One recent study found that 30-day readmission risk could be predicted with just ICD-9 and CPT codes, with an accuracy that met or exceeded eight existing models [7]. Therefore we felt CPT codes would be beneficial in our phenotyping algorithms. Unfortunately, because CPT is used for billing, codes are annually retired and replaced with new codes as insurance plans change. For example, in 2013, all outpatient psychology codes were replaced with new codes (with slightly different meanings). This can make development of phenotyping algorithms difficult. In our network, the Scalable Collaborative Infrastructure for a Learning Healthcare System (SCILHS), a group of clinical experts developed an algorithm to select a cohort of patients with a rare disease, Pulmonary Arterial Hypertension, using a combination of ICD-9 and CPT codes. However, as this was run at several hospitals, problems became apparent – several institutions had no patients that matched the criteria. One of the problems was that the clinicians’ algorithm did not include the deprecated CPT codes that had been removed from current usage but were still very common in historic data.

Terminology change management is always fraught, but it is particularly problematic with CPT codes. As discussed above, CPT changes, sometimes in major ways, annually. Furthermore, it is maintained by the American Medical Association and is not freely usable. Therefore, knowledge-based resources on CPT code management are not widely available. Medicare publishes major changes in CPT codes on an annual basis, but it is difficult to find information on changes prior to 2011 and the documents are not in computer-readable format. Commercial services do provide some documentation on deprecated CPT codes, but these are neither freely available nor are they comprehensive - most only go back a few years.

We sought to develop and evaluate a simple, free method to place deprecated CPT codes into the CPT hierarchy provided by the National Center for Biomedical Ontology (NCBO)’s BioPortal, a comprehensive repository of standard terminologies [8]. Our goal was not to exactly replace deprecated codes with new codes – codes are often replaced with code and modifier combinations that have slightly different meanings. Rather, we sought to insert codes correctly into the hierarchy so that hierarchy-aware phenotyping algorithms will correctly include current and deprecated codes that are children of the term used in the algorithm. A recent review of phenotyping algorithms found that using such code categories is one of the top “design patterns” found in Phenotype Knowledgebase (PheKB) algorithms [9]. Like many clinical data warehouses, Informatics for Integrating Biology and the Bedside (i2b2) clinical data analytics platform is hierarchy-aware. i2b2 is widely used for cohort selection in preparatory-to-research work [10], and it is used at over 100 sites nationwide and by over a third of PCORnet Clinical Data Research Network sites.

### CPT codes

Current procedural terminology (CPT) is the most common procedure terminology in the United States because it is almost ubiquitously required by billing systems. CPT provides five digit codes that identify specific services rendered (as opposed to purely clinical information, such as a diagnosis). CPT codes are arranged into six broad categories, and services like BioPortal provide more specific category groups for research and data analysis.

CPT is in its fourth edition, but as discussed previously, it changes every year. When codes are retired, they are not reused, which limits the number of new codes that can be introduced. However, retired codes are removed from the terminology. The CPT hierarchy is arranged to leave gaps between categories to leave room for new codes.

The codes represent very specific, detailed services that are carefully specified for billing purposes, such as the exact method used, the report types produced, and the types of results – anything that is relevant to reimbursement. CPT codes can be associated with multiple ‘modifier codes’ to further specify details of the procedure. Some code sets rely on modifiers for complete specification, while others insert their entire meaning into the code description. (CPT modifiers tend to have little clinical significance and are seldom used for research. Thus, they are beyond the scope of this manuscript.) Consequently, CPT code descriptions tend to be extremely complex and can be several sentences. For example, one allergy testing procedure code is described as “allergy testing, any combination of percutaneous (scratch, puncture, prick) and intracutaneous (intradermal), sequential and incremental, with drugs or biologicals, immediate type reaction, including test interpretation and report, specify number of tests.” There are allergy test codes for other billable variations of this code as well (e.g. intracutaneous-only allergy testing).

## Methods

### Design

For a current set of CPT codes, we imported the 2014AA release of CPT into i2b2 using the NCBO BioPortal import tool for i2b2 [11]. To identify the most salient deprecated codes, we examined codes in the Partners Healthcare Research Patient Data Registry (RPDR), an enterprise-wide research repository at Partners Healthcare. RPDR is accessible to Partners investigators and supplies data for the Partners SCILHS site. Because it has data on such a large patient population (>4.5 million patients), it is often used for preliminary data characterization in SCILHS. RPDR analysts pragmatically update their ontology annually, manually adding new codes that are seen in billing data. While this is not a comprehensive set of deprecated codes, we surmised that Partners’ CPT code list would include many of the deprecated codes used at other sites. We created a dataset of all patients seen at MGH or BWH since 2000 and imported this into i2b2, yielding 3,010,950 patients. Out of 8,262 CPT codes in this patient set, we found that 573 CPT codes were not present in 2014AA, which accounted for 6.5 million occurrences. We then developed a method to properly place the codes into the 2014AA CPT hierarchy.

### Related work and failed approaches

Ontology mapping approaches in medical informatics take two general forms: semi-automated and manual.

In practice, large-scale manual mapping is the most common endeavor, in which teams of consultants (such as Apelon or Intelligent Medical Objects) are employed to integrate terminologies. Because resources are limited in many informatics research projects, we did not explore this approach further.

Semi-automated approaches tend to utilize the terminology information provided by the Unified Medical Language System (UMLS), which provides many medical terminologies, their hierarchical structure, linkages between terminologies, and tools and thesarii to find terms in free text [12]. BioPortal provides resources similar to UMLS, with greater terminology breadth but lesser depth [13]. The UMLS MetaMap tools use Natural Language Processing (NLP) to normalize sentences and perform concept recognition, thereby matching phrases to concepts codes in standard terminologies [14]. A variety of other tools also combine these resources with NLP and are aimed at extracting medical concepts from free text [15], but for the most part such complex methods perform worse than simple approaches like lexical matching for ontology-to-ontology mapping [13]. Little has been published on ontology augmentation through mapping, and we found nothing on CPT augmentation. Papers that have been published tend to use UMLS or BioPortal in combination with a terminology-specific resource (e.g., Veterans Administration Drug Class mappings [16]). Therefore, we explored approaches involving: a) NLP, and b) terminology resources (UMLS, BioPortal, and CPT-specific). Both of these approaches failed.

### Natural language processing

Because no existing NLP tools were designed to recognize retired CPT codes, we performed preliminary analyses to assess the feasibility of an NLP approach. Our analyses consisted of measuring similarity between deprecated code descriptions and hierarchical grouper descriptions found in the 2014AA BioPortal CPT ontology. We explored the following approaches:

1. *Partial string matching* [17]*.* This is the simplest similatiry measure. We calculated the percentage of the string in the grouper’s description equal to the code description, and we used the top performer as a match. While this approach worked well in some cases (e.g., all of the debridement procedures begin with the word “debridement” and so did the appropriate grouper description), it failed when the word order was different between the two descriptions, which happened frequently. For example, “Tumor imaging, positron emission tomography (PET)” did not match to the category “Positron emission tomography (PET) imaging”.
2. *Bag of words.* Many Information Retrieval systems (such as simple search engines) treat two phrases as two bags of words (BOW) [18]. In BOW comparison, the percentage of words in common between two phrases is assesed, without regard to word order. Because the code descriptions are much more specific than the groupers, we used the grouper description as the target; we computed the percentage of words in the grouper that were also found in the code description. We assigned each code to the grouper having with largest percent match. This approach correctly found correlations where word order differed (an improvement over the previous method). However, both approaches discovered false correlations due to missing keywords. For example, “**Insertion** of epicardial single or dual chamber pacing cardioverter-defibrillator electrodes by thoracotomy” had the highest BOW match to “**Removal** of single or dual chamber pacing cardioverter-defibrillator electrode(s)”. (Notice the problematic boldfaced keyword.)
3. *Levenshtein distance* [19]*.* Returning to partial string matching, we computed the Levenshtein distance between the two descriptions. Levenshtein distance, also known as edit distance, counts the number of single letter edits that would be needed to convert the source string into the destination string. For example, the edit distance between “killing time” and “wasting time” is 4. In general, shorter edit distances mean that two strings are more similar. However, this approach proved little better than partial string matching, because the edit distance did not adapt well to word order/phrase order differences.

After these attempts, it became clear that the subtle variation in key words and parenthetical phrases would hinder any straightforward NLP approach. We discovered many of these keyword variations: insertion vs. removal, continuous vs. non-continuous, ‘other than clamp’ vs. ‘using clamp’. These would need to be disambiguated from unimportant words. Additionally, synonymous terms between the two descriptions would need to be reconciled and word order issues would need to be resolved.

We are aware that NLP tools exist which could assist in developing a complex NLP approach for this use-case. WordNet [20], which clusters semantically similar words, could be used to normalize synonyms. UMLS MetaMap could be used to discover medical concepts in the description. We are not aware of any available tools to rank the importance of words in procedure descriptions.

We decided to avoid NLP approaches because no simple NLP solution would be possible, and our background research previously indicated that complex NLP solutions for ontology mapping are frequently problematic [13].

### Terminology resources

We next attempted to place the deprecated codes into the hierarchy through terminology linkages in standard terminologies. We found the majority of codes in the Unified Medical Language System (UMLS) concept table, but the deprecated codes were not present in the 2014AA UMLS’ relationship table. Therefore no information on their relationship or hierarchical position was present. Seventeen CPT codes did fall into the same UMLS “lexical category” as a non-deprecated code, but lexical identifiers are not guaranteed to be stable year after year, so this did not seem to be a reliable method for mapping. The only other public terminology resource containing CPT that we found was the Observational Medical Outcomes Partnership’s Common Data Model (OMOP) vocabulary version 4.4, but again, no hierarchy information existed. OMOP does provide a CPT to SNOMED mapping that can be used to build a SNOMED-based procedures hierarchy, but it only included 56 of our deprecated CPT codes.

### Category matching approach

Since explicit linkages and lexical similarity did not succeeed, we developed a *numerical* similarity algorithm for category-matching CPT codes. To our knowledge, this approach has not previously been considered.

While Category I CPT codes are 5-digit, BioPortal provides 7-digit codes that represent higher-level groupings of codes, such as “cardiac catheterization procedures”. There are several levels of grouper codes. Importantly, CPT code groups are arranged numerically - each code is most similar to the codes numerically closest to it. So, for example, the codes closest to Caesarean section are lower-abdominal procedures. Slightly more distant are internal-organ procedures (such as kidney transplant) and even further are procedures on bones and joints.

The *hierarchcal* and *numerical similarity* properties allowed us to develop an algorithm to place codes with their most similar cousins in the hierarchy. This algorithm is presented in Table 1. The first portion of the algorithm creates a list of all groupers (*g*) in the 2014AA Bioportal CPT ontology, their hierarchical level (*l*), and the minimum (*a*) and maximum (*b*) code under each grouper. Then, the second portion finds the most specific grouper – where max(l) |a<d<b – for each deprecated code.

**Table 1:**
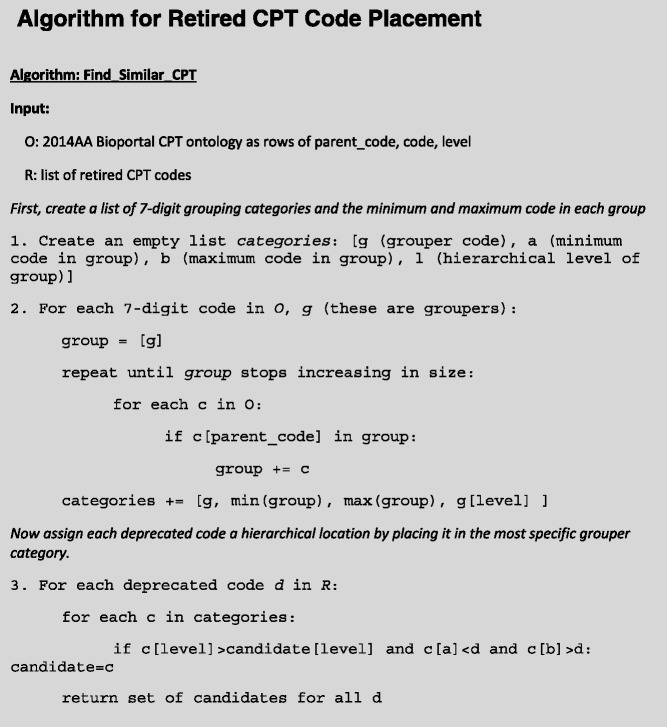
Algorithm to place retired codes into the 2014AA BioPortal CPT hierarchy using the property of numerical similarity. The algorithm first creates a list of all groupers (*g*) in the 2014AA Bioportal CPT ontology, their hierarchical level (*l*), and the minimum (*a*) and maximum (*b*) code under each grouper. Then, the the algorithm finds a grouper (*g*) where max(l)|a<d<b for each deprecated code *d*

An example of the algorithm is shown in Table 2. Step 3 of the algorithm sets *d* to 850. It then discovers three candidate rows in the list of grouper categories such that *a < d* and *b > d*: 100–1999, 800–882, and 840–851. The third row, 840–851, has the maximum hierarchical level (3), and so this row is assigned as the retired code’s grouper.

**Table 2:** Possible groupers for “Anesthesia for Caesarean section”

Groupers for Anesthesia for Cesarean section (850)			
l	a	b	Name
1	100	1999	Anesthesia
2	800	882	Anesthesia for procedures on the lower abdomen
3	840	851	Anesthesia for intraperitoneal procedures in lower abdomen including laparoscopy

The third (most specific) is chosen by our method. *l* is hierarchical level, *a* is the smallest code in this category, and *b* is the largest code

**Fig. 1:**
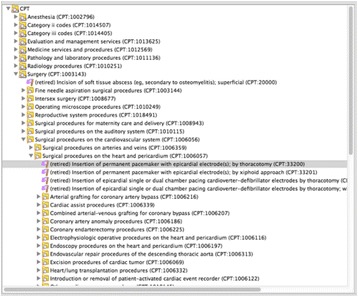
The i2b2 mapping tool, showing our algorithms’ suggested placements for retired CPT codes. This freely-available tool can be used to manually map, visually validate, and generate a merged i2b2 ontology file. In this study, we used this tool for some visual validation and to generate the ontology file. Our automated method created the mappings

We implemented this algorithm as a SQL script. This assigned categories to all deprecated codes. We then set up the results for manual verification by placing the mappings into the i2b2 Mapper tool, which allows graphical editing and verification of merged ontologies [21]. The Mapper tool with the CPT ontology and proposed placement of deprecated codes is shown in Fig. 1.

For validation, we chose the 171 codes that our method placed in the most specific possible grouping category, meaning that the category contains only CPT codes and not other groupers. We considered these the most potentially error-prone placements, because the categories are very narrow – therefore they are so specific that new codes might not make sense in that grouping. A clinician expert (AT) verified the location of these 171 deprecated codes in the CPT hierarchy. He was asked to verify the optimal position in the tree for the code and move the code if necessary.

Hierarchical classifiers’ performance is often subjective, because the meaning of the results is dependent on the required use-cases [22]. A variety of cost-estimate-based variants on precision and recall have been proposed, but the only agreed-upon approach is calculation against a gold standard [23]. Therefore, we considered the post-review validation set a preliminary gold standard. Then, we created a second gold standard by keeping only the reviewer’s changes to the validation set which were due to true errors. The criterion used was: “Is the classifier-placed parent folder actually a miscategorization, or is the reviewer’s change primarily cosmetic (e.g., mildly increased fidelity or a matter of opinion)?” This secondary analysis was performed by JGK. The first gold standard allows us to study the automated method vs. optimal placement (we will call this “optimality”), and the second supports detection of actual errors (we will call this “correctness”). We report non-hierarchical precision (PPV) on the results against both gold standards. We also provide descriptive statistics about the differences.

An informatician with experience in clinical informatics verified the remaining 402 codes, using the miscategorization criterion above. Because of the strictness of this criterion, all changes should have been due to miscategorization. However, the secondary analysis was still performed for verification.

Finally, we used the i2b2 Mapper tool to merge the verified ontologies (shown in Fig. 1). We then inserted the merged CPT tree into a PCORnet Common Data Model (CDM) representation in i2b2 that we are developing. This CDM ontology is being used in SCILHS currently, and three other PCORnet networks are presently adopting it [24].

### Deployment and evaluation

We deployed our augmented ontology to seven SCILHS sites, which implemented it against their data and provided us with a report of code coverage by our ontology. Secondarily, we evaluated the importance of the deprecated codes and our hierarchical approach in performing research. We did this by selecting several relevant and important phenotyping algorithms from our experience with SCILHS and from the literature. We examined whether the algorithms’ code lists contained both retired and non-retired codes. If so, we evaluated whether our augmented ontology contained both sets of codes. If not, we used the hierarchical ontology to find the codes’ retired siblings and quantified the additional patient population detected when the retired codes were included.

Finally, we utilized our ontology to visualize retired vs. non-retired codes. We selected relevant codes from the previously chosen algorithms, we used the hierarchical nature of the ontology to select all retired and non-retired sibings of those codes, and then we then plotted the number of occurences by year of each using Partners data.

## Results

### Date analysis

**Fig. 2:**
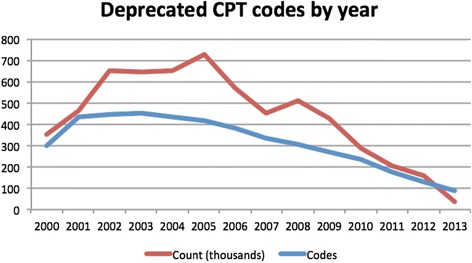
CPT codes present in RPDR but not in BioPortal CPT 2014AA, by year. *Count* is instances of the code, *Codes* is the number of unique codes

We analyzed the frequency of deprecated codes in the data, by year. The number of codes and their frequency is shown in Fig. 2.

### Validation set

The clinician expert made correctional moves to 82 of the 171 category-matched code placements to create the validation set. A breakdown of the changes is shown in Table 3. The secondary analysis found that 14 of those changes were due to a miscategorization. All four cases in which the clinician moved the code one level up in the hierarchy were miscategorizations – the classification was too specific. In 10 cases, miscategorization was discovered when the clinician moved the term into a parallel or distant folder, e.g., Rinkel test was miscategorized as an ingestion challenge test rather than an allergy test. Sixty-eight changes did not fix a miscategorization. In 51 cases, the clinician moved the code one level deeper in the hierarchy, which added further specialization. Twelve cases of moving the code into a parallel folder at the same level of the hierarchy were not miscategorizations. The parallel folders were minor variations of each other – e.g., several codes were moved from “Injection, anesthetic agent” to “Introduction/injection of anesthetic agent (nerve block), diagnostic or therapeutic procedures on the paravertebral spinal nerves and branches”. In the remaining 5 cases, the clinician moved the term further, but the move was to a similar folder in a different part of the hierarchy.

**Table 3:** Analysis of the miscategorization of our method for placing deprecated CPT codes in the hierarchy

Miscategorization Analysis of Retired CPT Code Placement				
	Validation set		Remaining codes	
Code movement (correction) by human reviewer	Moved for optimal placement	Moved due to miscategorization	Moved for optimal placement	Moved due to miscategorization
Deeper	51	0	0	0
Higher	4	4	2	2
Parallel folder	17	5	2	2
Distant	10	5	7	3
Total codes	171		402	
Precision	52 %	92 %	97 %	98 %
Avg. precision	52.0 % (optimal placement)/96.4 % (due to miscategorization)			

The table body shows how the human reviewer moved codes to correct miscategorizations. The first pair of columns is for the validation set, and the second pair covers all remaining codes. (These are shown separately because the validation set was subjected to a more rigorous analysis of optimal placement.) *Moved for Optimal Placement* includes all codes moved by the reviewer. *Moved due to Miscategorization* includes codes moved due to a true miscategorization, according to the secondary analysis. *Total codes* is all codes in each set (validation and remaining). *Precision* is the precision of the automated method for each subset of data. The final line shows the average precision in two cases: considering all moved codes (optimal placement) and considering errors only. The precision for optimal placement of remaining codes is not included in the averages; the second reviewer was not told to move codes for optimal placement, so this result would be invalid

### Remaining codes

The informatician moved 11 of the remaining 402 codes. A breakdown of these changes also appears in Table 3. Three were moved very far in the hierarchy (e.g., psychiatry instead of therapeutic injections), two were moved to a parallel subfolder, and two were moved up in the hierarchy. The remaining four codes were moved from the diagnostic & laboratory tree to the surgery tree. These were not errors, but it made the code placement consistent with other similar codes.

### Deployment

**Fig. 3:**
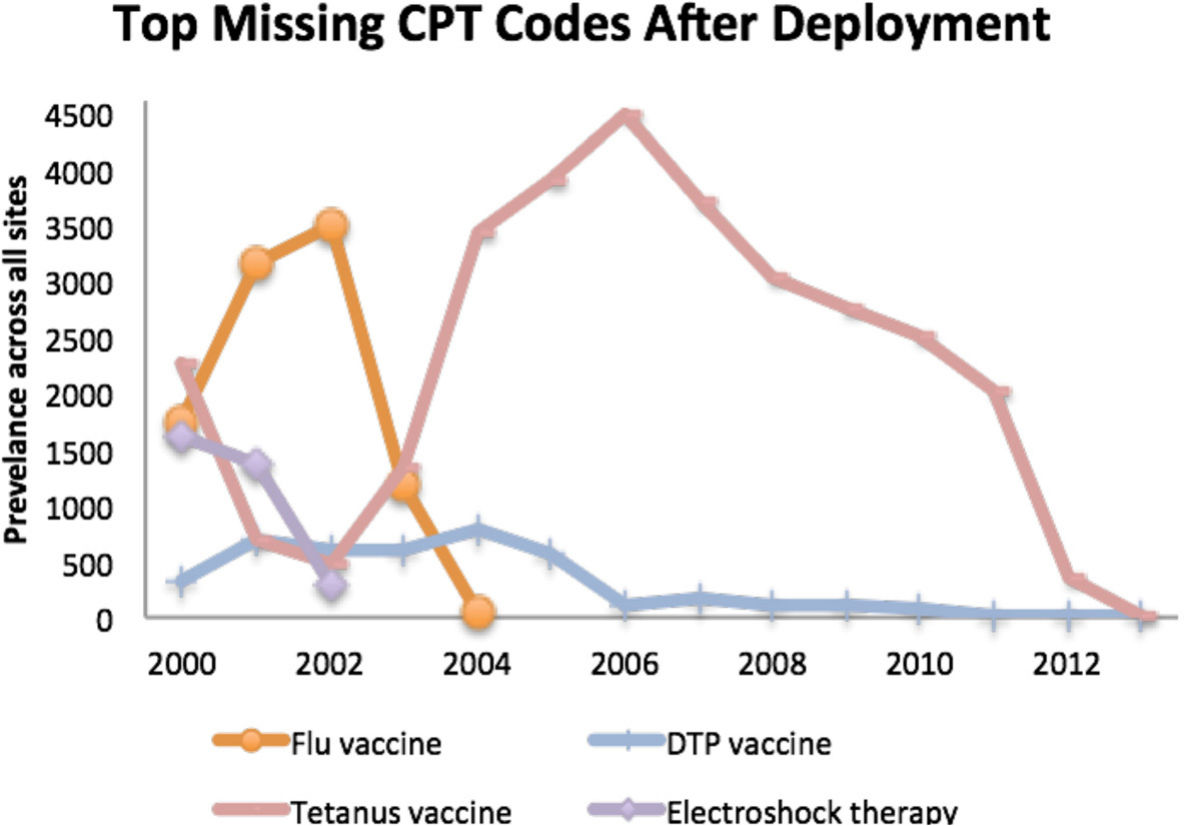
The top 4 CPT codes present at one or more of our seven sites but not in our augmented hierarchy. These account for 93 % of all missing instances across all sites. Most of the instances after 2004 are due to some sites using a different formulation of tetanus vaccine than Partners. This is also the only code used at multiple sites

We have deployed this new hierarchy to seven sites in the SCILHS network. Among these sites, 100 total CPT codes were missing from the augmented hierarchy, accounting for 51,695 instances. However, only 9 codes at four sites occurred more than 100 times. The four most frequent codes accounted for 93 % of all instances, including the only two codes with high prevalence that were used by multiple sites. These two codes were both for tetanus vaccine. A graph showing the temporal distribution of these top four codes across all sites is shown in Fig. 3. Three of these codes were for vaccines. We found that Partners’ facilities used slightly different formulations of these vaccines (with different CPT codes). The fourth, electroshock therapy, was always coded in RPDR as “single seizure per day,” which is a different CPT code than the missing one.

### Phenotyping improvement

We analyzed the impact of retired CPT codes on several relevant and important phenotyping algorithms. We found that PheKB [9], the phenotyping repository for the eMERGE network [25], uses CPT codes in 50 % (15/30) of its public phenotyping algorithms, most frequently to refine cohorts found through diagnosis codes. The impact of our ontology on phenotyping algorithms that use CPT codes in PheKB, SCILHS, and other literature fell into three categories as follows:

- **Algorithms do not include all current and retired codes: potentially high impact.** If our hierarchical approach correctly placed retired codes in the same grouper as the current code, a researcher could easily find both to update the algorithm. A salient example is SCILHS’ preliminary Pulmonary Arterial Hypertension algorithm, which considered right heart catheterization as an important diagnostic indicator. The code in the algorithm was retired at the end of 2010, so no recent patients were selected by this algorithm. Our ontology correctly placed the new and deprecated codes in the same categorical grouper (“Cardiac Catheterization Procedures”), making our phenotyping algorithm easily updatable. This was also true in many other cases, such as outpatient psychology procedures, for which (as mentioned in the Introduction) a significant code revamp occurred in 2012. Examples codes in these two categories are shown in Table 4. However, in some cases the augmented ontology placed codes at too general a level to be easily detected. For example, in the previously mentioned rehospitalization prediction study [7], retired codes for diagnostic injection were placed in the high level ‘Medicine Services and Procedures’ folder.
- **Algorithms include all possible codes: medium impact.** Our augmented ontology is still important in this case, because it allows access to data involving the retired codes. Without this, our SCILHS sites would not have implemented mappings to the retired codes. However, the hierarchical approach is not beneficial for algorithm maintenance in this case. (The hierarchical approach might still be useful to sites implementing the ontology, because local mappings could be streamlined by the grouping of similar codes.) We found this with the PheKB algorithms, which tend to have very complete code lists. For example, in their appendicitis algorithm, interventional radiology codes are used to refine the cohort. The published code list included both current and retired codes, and our augmented ontology did as well; without this, cases prior to 2007 would not be detected.
- **Algorithms do not involve codes that have changed: low impact.** Some phenotyping algorithms use CPT concepts that have not yet been replaced. We found this to be the case for rehospitalization detection in patients with chronic hepatitis [7]. In this case, the augmented ontology is not beneficial.

**Table 4:** Most frequent CPT Code descriptions for Psychiatric Evaluation and Right Heart Catheterization, among retired and new codes

Category	Status	Code description
Psychiatric evaluation	Retired	* Psychiatric diagnostic interview examination
		* Individual psychotherapy, insight oriented, behavior modifying and/or supportive, in an office or outpatient facility, approximately 20 to 30 min face-to-face with the patient
	Current	* Psychiatric diagnostic evaluation
		* Psychiatric diagnostic evaluation with medical services
		* Psychotherapy, 30 min with patient and/or family member
		* Psychotherapy, 30 min with patient and/or family member when performed with an evaluation and management service (list separately in addition to the code for primary procedure)
Right heart catheterization	Retired	* Right heart catheterization
		* Combined right heart catheterization and transseptal left heart catheterization through intact septum (with or without retrograde left heart catheterization)
	Current	* Right heart catheterization including measurement(s) of oxygen saturation and cardiac output, when performed
		* Catheter placement in coronary artery(s) for coronary angiography, including intraprocedural injection(s) for coronary angiography, imaging supervision and interpretation; with right heart catheterization

In Psychiatric Evaluation, notice the decreased complexity of the new codes (additional modifier codes are now used in conjunction with the main code). In Right Heart Catheterization, notice the movement of “right heart catheterization” to the end of the description in the second code. The actual CPT codes are not shown due to copyright restrictions

**Fig. 4:**
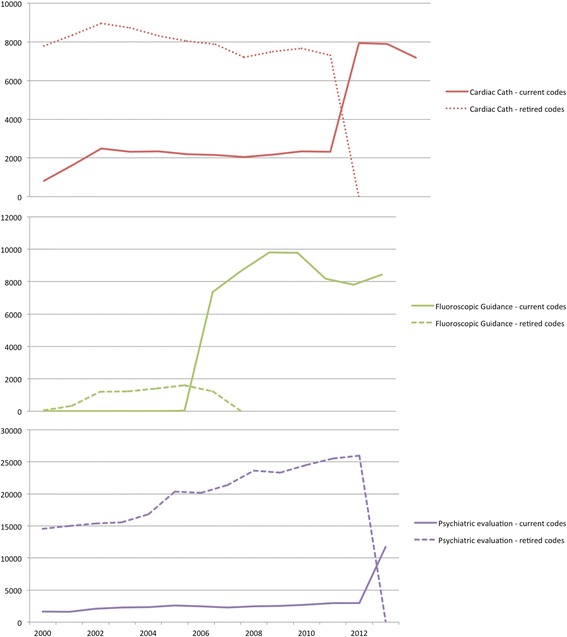
The volume of procedures for three categories of retired codes (dotted lines) vs. current codes (solid lines of the same colors). This visualizes the rapid drop-off of retired codes in transition years (2005,2010,2012)

Finally, our visualization of retired vs. non-retired codes can be seen in Fig. 4. We selected codes in three clinical areas inspired by our algorithm evaluation (cardiac catheterization, fluoroscopic guidance, and psychiatric evaluation). The Figure shows the number of occurences per year of all retired vs non-retired sibings of those codes. Notice the visiblilty of years where code transitions occurred (2010, 2005, and 2012, respectively).

## Discussion

Our method for hierarchical CPT code placement had 96.4 % precision when considering miscategorization only. In other words, 96.4 % of deprecated codes were placed in a position in the tree that did not result in miscategorization. Therefore, the automated method placed the vast majority of codes in a hierarchical position that will support research and hierarchy-aware queries. Moreover, the precision is high enough that the informatician verifier was able to cursorily review the codes very quickly. The caveat is that cursory review is still necessary, because the remaining 3.6 % of codes were actually miscategorized.

According to the validation set, our method had only 52 % precision when considering all of the differences between the classifier and gold standard. In other words, the deprecated codes were placed in an optimal position only 52.0 % of the time. While this is clearly non-optimal, we found it sufficient for our SCILHS work because only 8 % of the changes were due to errors, and the other 92 % would not generally impact the use-case of finding deprecated codes’ current siblings.

The informatician reviewer found a lower error rate than the clinician (2 % vs 8 %). This could be due to lack of expertise or the more cursory review of codes, but we suspect it is because fewer errors actually occurred when codes were placed in less specific categories, because the groupers are broader.

The seven sites that have implemented our hierarchy had only a minority of missing codes (9 with prevalence >100), and only two of these missing codes were used at multiple sites. Therefore it is unlikely that the missing codes would be useful in cross-site queries. This analysis gives us some confidence that the merging of the RPDR and 2014AA BioPortal CPT tree covers nearly all CPT codes that will be used in research. This hierarchy will be used in our SCILHS network for forthcoming phenotyping algorithms.

Our survey of phenotyping algorithms found that CPT is important in existing algorithms (including 50 % of public algorithms in PheKB), and our augmented ontology often correctly pairs retired codes with non-retired codes when the algorithms’ code list is incomplete (e.g., for right heart catheterization and outpatient psychiatry). Additionally, it enables helpful visualizations of frequency of retired codes by year, which could be useful for quality-assurance in algorithm development. Even when the developed algorithm’s code list is already comprehensive, the augmented ontology contained the retired codes, enabling research using older data. In a few cases, the algorithm did not correctly pair retired and non-retired codes (due to very general placement of siblings). This limitation is part of the reason for the present manual verification step.

This category-matching methodology was implemented as a SQL script, and this research was built on freely available tools. It is directly implementable in the popular i2b2 platform, but the approach is also applicable for any ontological approach to CPT representation (such as the NCBO BioPortal). Because CPT *itself* cannot be widely distributed due to copyright restrictions, it is important to develop freely available *methods* to augment individual groups’ licensed CPT hierarchies. Finally, because the approach involves only code matching, it scales very well computationally. The manual verification step is a bottleneck at high scale, but the looser second-level evaluation (correctness vs. optimality) took the informatician only a few hours with almost 600 codes. This is much less resources-intensive than the manual mapping services offered by major commercial entities.

The overall SCILHS / PCORnet data model is available on the SCILHS website [26]. This public release does not contain CPT codes due to copyright restrictions, but we are happy to distribute our ontology to anyone that has a CPT license. Please contact SCILHS for more information. Likewise, the SQL script for category matching is available upon request from the authors.

## Conclusion

We have developed a straightforward method using freely available tools to place deprecated CPT procedure codes into the hierarchy provided by BioPortal. No existing published approach or dataset concerns itself with *hierarchical placement* of deprecated CPT codes, which is important in phenotyping algorithms. This approach placed codes with 96.4 % precision when considering miscategorization only, which allowed rapid verification by medical informatics experts. Codes were placed with only 52 % precision against a gold standard of optimal categorization, because codes were not always placed in the most specific possible sub-folder. However, this will not disrupt development of many phenotyping algorithms, because the most common use-case is to find deprecated codes’ current siblings. We have implemented this augmented CPT tree in i2b2 and deployed it at seven sites in our network. Ths CPT ontology is available by request to SCILHS for holders of CPT licenses. Only a handful of codes were missing from our hierarchy in SCILHS, and 93 % of missing code instances were due to just four codes (flu vaccine, tetanus vaccine, and electroshock therapy). Among missing codes, only the tetanus vaccine codes were used at multiple sites, meaning the missing codes would be of limited utility for cross-network querying. Our survey of phenotyping algorithms indicated our approach benefits the development and implementation of these algorithms, and in particular the hierarchical nature allows straightforward algorithm updating and performance monitoring as codes and retired and replaced.

## Abbreviations

AHRQ: Agency for Healthcare Research and Quality
BWH: Brigham and Women’s Hospital
CDM: Common Data Model
CPT: Current procedural terminology
EHR: Electronic health record
eMERGE: "Electronic Medical Records And Genomics" Network
i2b2: Informatics for integrating biology and the bedside
ICD-9: International classification of diseases, version 9
MGH: Massachusetts General Hospital
NCBO: National Center for Biomedical Ontology
NLP: Natural Language Processing
NQF: National Quality Forum
OMOP: Observational medical outcomes partnership
PCORI: Patient Centered Outcomes Research Institute
PCORnet: Patient Centered Outcomes Research Network
PheKB: Phenotype knowledgebase
PSI: Patient safety indicator
RPDR: Research Patient Data Registry
SCILHS: Scalable, Collaborative Infrastructure for a Learning Health System
SNOMED: Systmatized nomenclature of medicine
SQL: Structured query language
UMLS: Unified medical language system
